# How Many Loci Does it Take to DNA Barcode a Crocus?

**DOI:** 10.1371/journal.pone.0004598

**Published:** 2009-02-25

**Authors:** Ole Seberg, Gitte Petersen

**Affiliations:** Laboratory of Molecular Systematics, Natural History Museum of Denmark, Copenhagen, Denmark; Trinity College Dublin, Ireland

## Abstract

**Background:**

DNA barcoding promises to revolutionize the way taxonomists work, facilitating species identification by using small, standardized portions of the genome as substitutes for morphology. The concept has gained considerable momentum in many animal groups, but the higher plant world has been largely recalcitrant to the effort. In plants, efforts are concentrated on various regions of the plastid genome, but no agreement exists as to what kinds of regions are ideal, though most researchers agree that more than one region is necessary. One reason for this discrepancy is differences in the tests that are used to evaluate the performance of the proposed regions. Most tests have been made in a floristic setting, where the genetic distance and therefore the level of variation of the regions between taxa is large, or in a limited set of congeneric species.

**Methodology and Principal Findings:**

Here we present the first in-depth coverage of a large taxonomic group, all 86 known species (except two doubtful ones) of crocus. Even six average-sized barcode regions do not identify all crocus species. This is currently an unrealistic burden in a barcode context. Whereas most proposed regions work well in a floristic context, the majority will – as is the case in crocus – undoubtedly be less efficient in a taxonomic setting. However, a reasonable but less than perfect level of identification may be reached – even in a taxonomic context.

**Conclusions/Significance:**

The time is ripe for selecting barcode regions in plants, and for prudent examination of their utility. Thus, there is no reason for the plant community to hold back the barcoding effort by continued search for the Holy Grail. We must acknowledge that an emerging system will be far from perfect, fraught with problems and work best in a floristic setting.

## Introduction

The primary aims of DNA barcoding are to identify known specimens and to help flag possible new species, thereby making taxonomy more effective for science and society (www.barcoding.si.org).

The majority of DNA barcoding studies in animals have used a small region of the mitochondrial gene, cytochrome c oxidase subunit I (COI), as barcode, and COI is promising, with a few exceptions [1], to be the universal barcode for animals [2]. The search for a universal barcode in plants has been far more tortuous, but general agreement is emerging that more than one region is needed [3]–[7] (for a dissenting view; [see 8]), and that these regions will need to come from the plastid genome, where several different regions have been suggested. The plant mitochondrial genome is usually far too conservative to be of use [3]–[6], [9], whereas the plastid genome shares several of the desirable properties found in the animal mitochondrion. The nuclear multi-copy internal transcribed spacers array (ITS) has also been suggested as a barcode [3], [4], but has been discarded due to its peculiar pattern of evolution [10] and currently the nuclear genome is largely inaccessible for barcoding purposes. However, current advances in sequencing technology and the diminishing expenses promise radically to change the way we do barcoding; [see e.g. 11]. The majority of plastid regions have been proposed on the basis of comparisons of levels of variation in whole genomes of closely related taxa, e.g. congeneric species, and subsequently tested in taxonomically widely dispersed, randomly chosen species pairs [3], [4], [12] and/or in a purely floristic setting [8], [13]. As a consequence, emphasis is placed on primer universality at the expense of species recognition; see however Fazekas et al. [7].

Chase et al. [3], using data predominantly drawn from GenBank, and acknowledging the imperfect nature of these data, suggested the use of one or two, unspecified, plastid regions plus ITS as potentially useful plant barcodes. Almost simultaneously Kress et al. [4] pointed to the potential value of a combination of ITS and the *trnH-psbA* spacer. The choice of the latter region was based on a combination of comparisons of the complete plastid genomes of deadly nightshade (*Atropa belladonna* L.) and tobacco (*Nicotiana tabacum* L.), and tests on a small, taxonomically defined set of species and a larger one defined geographically. Using a similar approach, but restricting the search to coding plastid regions only, Chase et al. [14] selected a number of potentially useful regions (see www.kew.org/barcoding) that subsequently were tested by a number of research groups worldwide on a limited taxon set. Based on an evaluation of their overall performance in these tests, a smaller set was chosen subsequently, and these favoured regions tested more widely, and two sets were proposed eventually as universal barcodes, *rpoC1* and *matK* plus either *rpoB* or *trnH-psbA*. However, these two triplets were challenged by Kim et al., who preferred a combination of *matK*, *atpF-H*, and *psbI-psbK* [15].

On the basis of its ability to distinguish between congeneric species-pairs, Newmaster et al. [5] proposed *rbcL* as the first tier in their proposal of a two-tiered approach to barcoding; allowing the next region to be an optional choice. Kress and Erickson [12] refined this proposal by suggesting only the 3′ end of *rbcL* as their first choice and adding *trnH-psbA* as their preferred second choice, again basing their conclusion on tests involving congeneric species-pairs. However, using a relatively dense taxonomic coverage (35% (mobot.mobot.org/W3T/Search/vast.html) of known species in a single genus), Newmaster et al. [5], while maintaining their choice of *trnH-psbA*, replaced *rbcL* with *matK*. This conclusion was supported by Lahaye et al. [8] on the basis of geographically defined, purely floristic and similarly designed but broader taxonomic studies in a test of all regions suggested by Chase et al. [14].

Acknowledging the low resolution of the *trnL* intron, even in a floristic context, Taberlet et al. [13] none the less suggested its use as a plant barcode, primarily due to the capability of its P6 loop to distinguish species in highly degraded or processed material. Based on a taxonomic study with the hitherto densest taxon sampling, 48% of all 278 known species of a single genus, Edwards et al. [16] suggested that at least three regions were necessary to discriminate most of the studied species, including a new region *trnT-trnL* in combination with ITS and “at least one more region with a greater level of variation than *psbA-trnH*” [16].

Even COI [7] and 23S rDNA [17] have been suggested as barcodes for plants, but have both been proven too invariable to be of general use [7].

## Results and Discussion

Here we present the first analysis of the performance of one of the recently proposed barcode sets by Chase et al. [14], *rpoC1*, *matK*, and *trnH-psbA*, in a large, taxonomically defined, monophyletic group; the genus *Crocus* L. (Iridaceae). We tested these regions on 86 (98%) species of the genus *Crocus*, excluding only two species of doubtful taxonomic status. To this set of regions we have added three other regions, two of which have been considered of potential value as barcodes (*rps8-rpl36* [4] and *accD* [14]), plus *ndhF*, which we have used for phylogenetic purposes [18]. The taxon sampling was not designed to capture intraspecific variation, but 17 species include more than one accession each (from 2 to 15). Though few studies of plants take intraspecific variation into account, this may be a bigger problem than generally believed, by reducing the barcode gap and consequently the success rate of identification [see 7].

The proposed barcode set [14] is diagnostic for 63 (73%) of the included *Crocus*-species, which is only marginally better than the combination *matK* and *psbA-trnH* alone, which identifies 62 (72%) species (Table 1). Substituting *rpoC1* with any of the two other considered regions, *accD* or *rps8-rpl36*, improves species recognition to 67 (78%) and 65 (76%), respectively. Using the four most variable of these above-mentioned regions (*ndhF*, *matK*, *trnH-psbA*, and *rps8-rpl36*) makes it is possible to identify 79 species (92%), a figure that is not changed by adding the two least variable regions (*accD* and *rpoC1*). In all instances the species level resolution is higher than the ones obtained by Fazekas el al. [7] using a similar number of loci. This is undoubtedly due to our non-tree based approach, which does not require gene-tree monophyly [7]. Interestingly, *ndhF*, which has not been suggested as a barcode due to lack of primer universality, has a higher resolving power than *matK*, which is one of the top candidates as a universal barcode. However, in most instances some regions will perform better than others no matter which are chosen as barcodes [7]. In general, identification success was not influenced by the inclusion of length variation. It is worth mentioning that the two alternative regions proposed by Kim et al. at the Consortium for the Barcode of Life meeting in Taipei, in addition to *matK*, do not behave, in a smaller subset of *Crocus* species (see Tables S1 and S2), better than any of those suggested by Chase et al. [14].

**Table 1:** Sequence variation and species identification ability of six plastid regions in combinations in 86 species of *Crocus*.

Regions	Unique species (%)
*ndhF*+*matK*	69 (80%)
*ndhF*+*trnH-psbA*	66 (77%)
*matK*+*trnH-psbA*	62 (72%)
*ndhF*+*matK*+*trnH-psbA*	75 (87%)
*matK*+*trnH-psbA*+*accD*	67 (78%)
*matK*+*trnH-psbA*+*rps8-rpl36*	65 (76%)
*matK*+*trnH-psbA*+*rpoC1*	63 (73%)
***ndhF*** **+** ***matK*** **+** ***trnH-psbA*** **+** ***rps8-rpl36***	**79 (92%)**
*ndhF*+*matK*+*trnH-psbA*+*accD*	77 (90%)
*ndhF*+*matK*+*trnH-psbA*+*rpoC1*	75 (87%)
*ndhF*+*matK*+*trnH-psbA*+*rps8-pl36*+*accD*+*rpoC1*	79 (92%)

The best combination, i.e., the combination of the fewest number of sequences yielding the highest number of identified species, is marked in bold.

**Table 2:** Sequence variation and species identification ability of six plastid regions individually in 86 species of *Crocus*.

Region	Aligned length	Variable sites		Variable sites (excl./incl.gaps)/aligned length	Unique species (%)
		excl. gaps	incl. gaps		
*ndhF*	769	141	147	18/19%	61 (71%)
*matK*	842	157	168	19/20%	49* (58%)
*trnH-psbA*	698	57	157	10/22%	42 (49%)
*rps8-rpl36*	554	53	102	10/18%	29 (34%)
*accD*	367	37	37	10/10%	18 (21%)
*rpoC1*	575	29	29	5/5%	11 (13%)

* Of 85 species. DNA was no longer available for *C. hartmannianus* Holmboe.

With the notable exception of *rbcL*, most of the plastid regions that have been suggested as official barcodes, and hence potentially being the most variable regions, stems from the same limited region, covering approximately ∼15% of the large single-copy region of the plastid, spanning from *rpoB* to *trnH-psbA*. Hence, it is not surprising that “there are multiple multilocus plant DNA barcoding combinations that perform about equally well in resolving species” [7].

**Figure 1:**
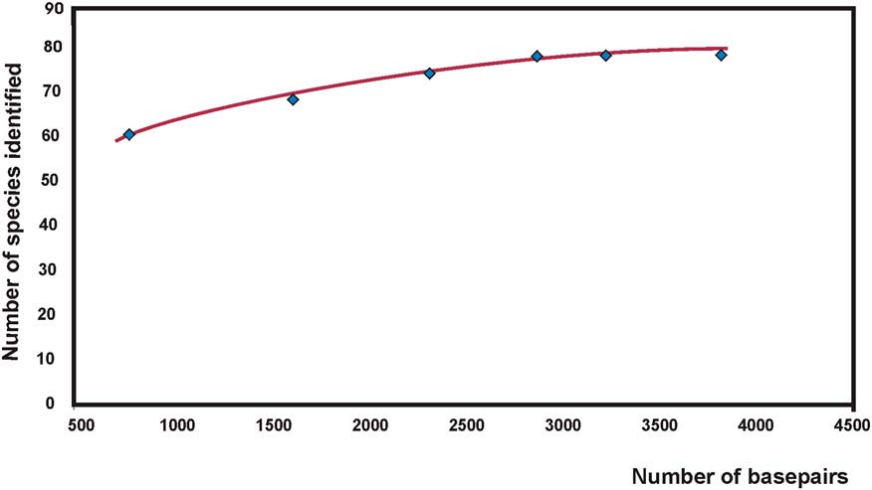
The relationship between sequence length and the number of *Crocus* species identified. The genus includes 86 known species, and the six regions (five proposed barcode regions plus *ndhF*) used here identify 79 species. The regions are added according to their ability to identify species. The performance of individual genes is shown in Table 2. The logarithmic trend line (y = 12.3 ln(x)−21.0) and the R^2^ ( = 0.97) were calculated in Excel® and checked in JMP, Version 7. SAS Institute Inc., Cary, NC, 1989–2007.

In figure 1 the relationship between the number of *Crocus* species identified is plotted as a function of the number of basepairs sequenced and the best fitting curve is added. Solving the equation for y = 86 (the maximum number of species) gives x = 5859, and it appears reasonable, at least in theory to postulate that it would require approximately 5800 bp from the plastid genome to identify all known species of *Crocus*. This corresponds to 9–10 average-sized (∼600 bp) barcode genes/regions and is presently not a workable option. Using a differently defined taxon sampling and a different criterion for correct identification a similar relationship between number of barcode regions and discrimination ability was found by Fazekas et al. [7].

Also, it would be problematic to flag potentially new species using these data because several species, most notably *C. biflorus* Mill. and *C. reticulates* Steven ex Adams, have non-monophyletic sets of plastid genomes (see figure S1). Disagreement between the evolutionary histories of organellar and nuclear genomes are not uncommon in plants ([see e.g. 19]) and animals [20], and it is a controversial point whether reciprocal monophyly [21] is a necessary requirement in barcoding. However, if reciprocal monophyletic species are mandatory this is bound to decrease identification success; see e.g. Fazekas et al. [7]. Using the same approach as Fazekas et al. [7] and a bootstrap cut-off value of 70% only two of the 17 species represented by more than one accession in the present analysis are monophyletic and two paraphyletic.

**Figure 2:**
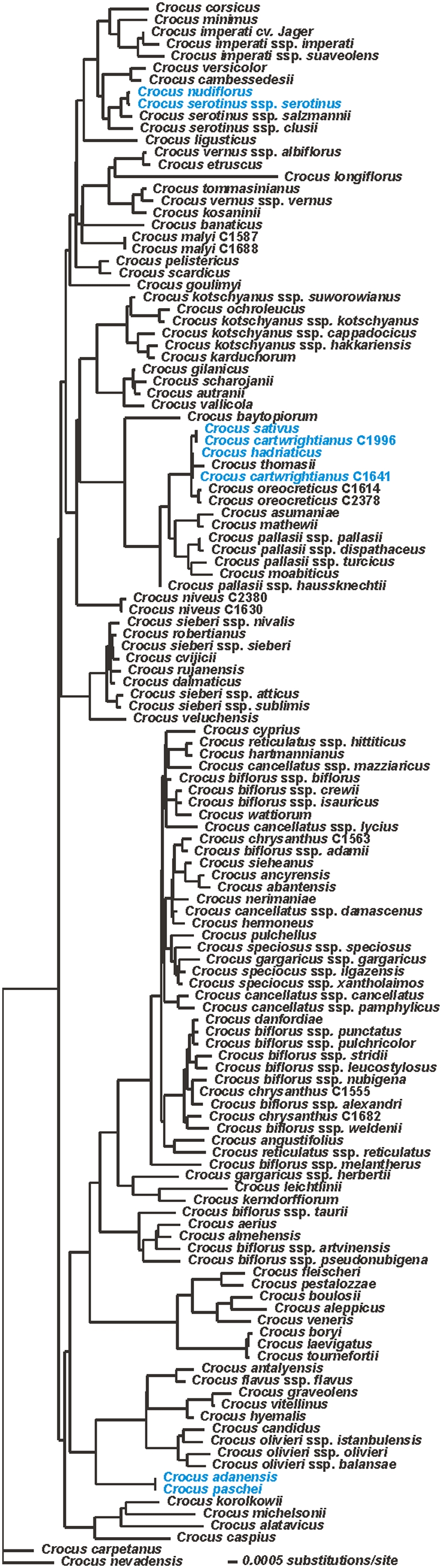
Neighbor-joining tree of *Crocus*. The extremely short branches make identification by cluster memberships difficult as does the “non-monophyly” of several species. Species that cannot be identified by any of the sequences used are marked in blue. The NJ tree is rooted with the two *Crocus* species that are sister group to the remaining *Crocus* species in the parsimony based phylogeny (see figure S1).

In a barcoding context (e.g. Barcode of Life Data System (BOLD); www.boldsystems.org [22]), identification is often conducted by reference to clusters of taxa in a neighbor-joining (NJ) tree using an average Kimura-2-parameter model. If this approach is used on the *Crocus* data set, many of the branch lengths are extremely short, making species assignment of new accessions spurious at best (figure 2). To aggravate this, a whole suite of NJ trees may be produced from the same dataset, due to the known input-order sensitivity of NJ. Resampling techniques are occasionally used to justify species monophyly [see e.g. 7], [8], [23] or to justify species identification; see e.g. Fazekas et al. [7]. Due to their innate properties [24]} and their very different implementation in different computer programmes [25] the use of resampling in this context is ill-advised, and would have made little sense here.

As in any other group of species, species circumscription in *Crocus* may be problematic, and this may be one factor underlying the observed lack of resolution. However, there is no reason to believe that taxonomic problems are more severe in *Crocus* than in many other taxa. Even in groups with an almost universally agreed upon species-level taxonomy, e.g., barley (*Hordeum* L.) with 32 species, it is impossible to recognise more than approximately 50% of the species using *matK* and *rpoC1* (see Table S3). Perhaps the worst case scenario is found among the morphologically distinct species of the Galápagos sunflower tree, *Scalesia* Arn.(Asteraceae), where no variation has been found (see Table S4) in the plastid markers, and almost none in nuclear markers.

Barcode efforts in plants are severely hampered by an obvious lack of agreement about the choice of regions. Opinions are divided on whether two or three regions suffice, whether regions should be coding or non-coding, whether one should or should not use a tiered approach, and ultimately on which regions are to be preferred and how the data are analysed, and the baseline for comparison has been ill-defined [8], [26]–[28]. In the most thorough comparison of all suggested regions to date, both with respect to technical and variability issues, it is recommended that three (or even four) regions, one coding (*rbcL*, *rpoB*, *matK*) and two non-coding (*trnH-psbA*, *atpF-atpH*), are selected as official barcodes [7] as this represents a reasonable compromise between effort and species resolution as it is currently implemented in the Barcode of Life Data System.

Both the present study and the study by Fazekas et al. [7] show that there is a limit to resolution no matter which region or regions are chosen. The present study shows that in a taxonomic setting and with a reasonable effort it is unlikely that barcoding will enable us to identify more than around 70–75% of the known species – in some instances less, in others more.

However, the time is ripe for selecting barcode regions in plants, and for prudent examination of their utility. Based on the level of sequence variation alone, an optimal set of regions is not yet known, but *matK* and *trnH –psbA* are strong candidates, though other conditions much notably primer universality and sequence quality have to be taken into account. However, we must acknowledge that the emerging system will be far from perfect ([see e.g. 29]), and that it will work best in a floristic setting.

Thus, there is no reason for the plant community to hold back the barcoding effort by continued search for the Holy Grail [30].

## Materials and Methods

### Taxon sampling

Taxon sampling was as extensive as possible. Of the 81 species recognized by Mathew [31] all but one, *Crocus boissieri* Maw, known only from a herbarium specimen, are included, as are six of the seven species described since then. Of the 50 recognized subspecies [31] 48 are included, but only two of the 10 later described ones. A total of 17 species were represented by more than one accession, and the total number of included accessions of *Crocus* is 131. Two species of *Romulea* Maratti, and one species each of *Syringodea* D. Don, *Babiana* Sims, and *Tigridia* Juss. were included as outgroups. Voucher information and GenBank accession numbers may be found in Petersen et al. [18].

### Molecular methods

DNA extractions were performed using the DNeasy Plant Mini Kit (QIAGEN Ltd., Crawley, West Sussex) after tissue disruption in a FastPrep FP-120 bead mill (Qbiogene, Carlsbad, CA). PCR amplifications followed standard procedures except for the addition of 0.1 mg/ml BSA to most reactions. For PCR amplification and sequencing of the five plastid regions the following primers were used: ndhF1318F and ndhF2110R [32], accD1F and accD3R (http://www.kew.org/barcoding/protocols.html), rpoC1F and rpoC4R (http://www.kew.org/barcoding/protocols.html), rpl36F and rps8R [33], and psbAF and trnH2 [34], [35]. Direct sequencing of purified PCR products was performed using BIGDYE 1.1 (Applied Biosystems, Wellesley, Massachusetts, USA) and purified sequencing products were run on an AB3130xl automated sequencer (Applied Biosystems). Sequence editing was done using Sequencher versions 4.5 to 4.7 (Gene Codes Corporation, Ann Arbor, Michigan, USA).

### Sequence analysis

In order to assess the potential value of the six sequence regions as barcodes, the outgroup taxa and the hybrid taxon *C.×jessopae* Bowles were excluded. All variable sites were included, and so was an ambiguously aligned region of *trnH-psbA* previously excluded from phylogenetic analysis. PAUP*, version 4.0b8 [36] provided numbers of variable sites. The number of uniquely identifiable species was checked using MacClade version 4.08 [37]. A species is considered uniquely identifiable if all the included specimens/subspecific taxa can be identified. Thus, species monophyly is *not* a requirement. Alignments were done manually and the matrix is available at TreeBase (acc no. M3519, S1912).

### Tree-based analysis

All phylogenetic analyses were performed using PAUP*, version 4.0b8 [36]. Uninformative characters were excluded from the phylogenetic analyses, and informative characters were equally weighted and treated as unordered. Gaps were treated as ambiguous data (?). Analyses were performed using both the default branch collapsing rule (collapse if maximum is zero) and amb- (collapse if minimum length is zero). The latter option was used for facilitating comparison of results from phylogenetic analyses using PAUP* with result from analyses using WinClada [38]. Under the default branch collapsing rule and simple sequence addition the number of equally parsimonious trees was very high (hitting the limit of 637.000 defined by memory allocation) and analyses without an upper limit for the number of saved trees could not be run to completion. Thus, we also used a two step approach first running 1.000 random addition sequences saving no more than 25 trees per replicate. The trees saved in this analysis were used as starting trees for a new analysis with a maximum number of trees saved set to 100.000. Phylogenetic analyses performed using WinClada, version 1.00.08 [38], spawning the matrix to NONA version 2.0 [39] were executed using heuristic search options hold10000, mult*100, max*, hold/10, and the default branch collapsing rule, amb-.

Neighbor-joining was also done in PAUP 4.0b8, using the default settings and Kimura-2-parameter distance option.

## Supporting Information

Figure S1 Strict consensus tree of Crocus and five outgroup taxa.
(6.33 MB TIF)

Table S1 Sequence variation and species identification ability of eight plastid regions in Crocus series Crocus. Crocus series Crocus is monophyletic (see figure S1) and includes nine species (C. sativus L., C. cartwrightianus Herb., C. hadriaticus Herb., C. thomasii Ten., C. oreocreticus B. L. Burtt, C. asumaniae B. Mathew & T. Baytop, C. mathewii Kernd. & Pasche, C. pallasii Goldb., C. moabiticus Bornm. & Dism. ex Bornm). Three species (C. sativus, C. cartwrightianus, C. hadriaticus) cannot be identified be any sequence. The length of the region atpF-H is 570–572 bp (573 bp in alignment). atpF-H GenBank acc. nos. EU523361-EU523373. The region psbI-K is very short (ca. 173–179 bp), but difficult to sequence due to several longer runs of T's (at least 3 runs of 9–10 or more T's).
(0.03 MB DOC)

Table S2 Sequence variation and species identification ability of six plastid regions in various combinations in Crocus serie Crocus. See Table S1
(0.03 MB DOC)

Table S3 Sequence variation and species identification ability of two plastid regions in Hordeum (all 32 species). No length variation is observed among the sequences. GenBank acc. nos. EU118371-EU118422, EU118427-EU118478.
(0.04 MB DOC)

Table S4 Sequence variation and species identification ability of six plastid regions in Scalesia (5 of 15 species). No length variation is observed among the sequences. GenBank acc. nos. EU118423-EU118426, EU118479-EU118483, EU118494-EU118498, EU118509-EU118513, EU118524-EU118527, EU118536-EU118539.
(0.04 MB DOC)
