# Supplementary figures and images for: How Many Loci Does it Take to DNA Barcode a Crocus?
Source: PLoS One. 2009 Feb 25;4(2):e4598. doi: 10.1371/journal.pone.0004598 (PMC2643479; doi:10.1371/journal.pone.0004598)

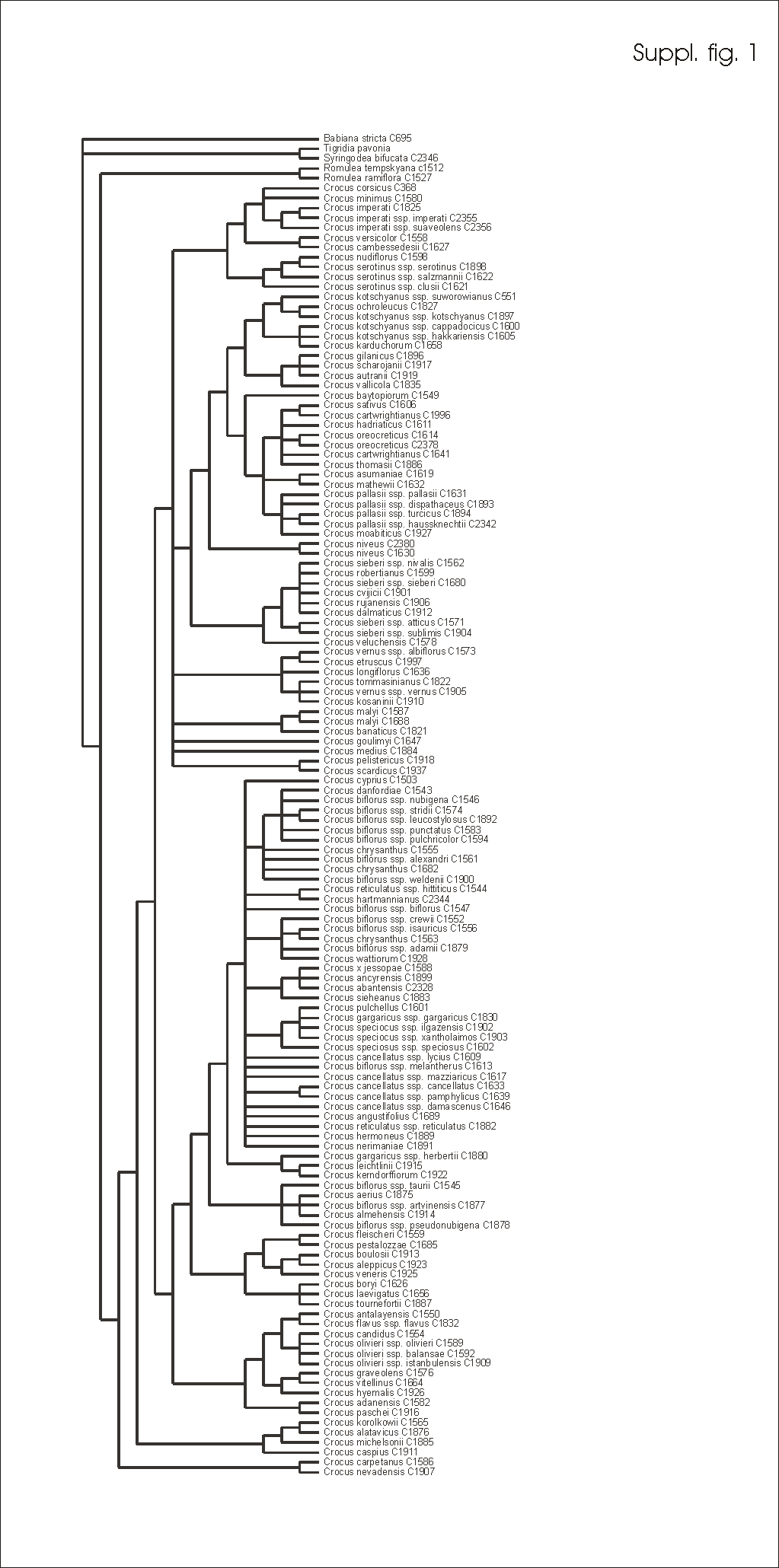

Supplement: Figure S1 — Strict consensus tree of Crocus and five outgroup taxa. (6.33 MB TIF) [file pone.0004598.s001.tif]
