# Supplementary material for: How Many Loci Does it Take to DNA Barcode a Crocus?
Source: PLoS One. 2009 Feb 25;4(2):e4598. doi: 10.1371/journal.pone.0004598 (PMC2643479; doi:10.1371/journal.pone.0004598)
Supplement: Table S1 — Sequence variation and species identification ability of eight plastid regions in Crocus series Crocus. Crocus series Crocus is monophyletic (see figure S1) and includes nine species (C. sativus L., C. cartwrightianus Herb., C. hadriaticus Herb., C. thomasii Ten., C. oreocreticus B. L. Burtt, C. asumaniae B. Mathew & T. Baytop, C. mathewii Kernd. & Pasche, C. pallasii Goldb., C. moabiticus Bornm. & Dism. ex Bornm). Three species (C. sativus, C. cartwrightianus, C. hadriaticus) cannot be identified be any sequence. The length of the region atpF-H is 570–572 bp (573 bp in alignment). atpF-H GenBank acc. nos. EU523361-EU523373. The region psbI-K is very short (ca. 173–179 bp), but difficult to sequence due to several longer runs of T's (at least 3 runs of 9–10 or more T's). (0.03 MB DOC) [file pone.0004598.s002.doc]

| **Region** | **Variable sites**  (incl. gaps) | **Unique species**  (%) |
| --- | --- | --- |
| *ndhF* | 11 | 4 (44%) |
| *matK* | 11 | 4 (44%) |
| *trnH-psbA* | 4 (14) | 2 (22%) |
| *rps8-rpl36* | 2 | 1 (11%) |
| *accD* | 0 | 0 (0%) |
| *rpoC1* | 3 | 2 (22%) |
| *atpF-H* | 4 (7) | 3 (33%) |
| *psbI-K* | ? | ? |
