# Supplementary material for: How Many Loci Does it Take to DNA Barcode a Crocus?
Source: PLoS One. 2009 Feb 25;4(2):e4598. doi: 10.1371/journal.pone.0004598 (PMC2643479; doi:10.1371/journal.pone.0004598)
Supplement: Table S2 — Sequence variation and species identification ability of six plastid regions in various combinations in Crocus serie Crocus. See Table S1 (0.03 MB DOC) [file pone.0004598.s003.doc]

| **Regions** | **Unique species**  (%) |
| --- | --- |
| *matK*+*ndhF* | 6 (66%) |
| *matK*+*trnH-psbA* | 6 (66%) |
| *matK*+*atpF-atpH* | 6 (66%) |
| *ndhF*+*atpF-atpH* | 6 (66%) |
| *ndhF*+*trnH-psbA* | 4 (44%) |
