# Supplementary material for: How Many Loci Does it Take to DNA Barcode a Crocus?
Source: PLoS One. 2009 Feb 25;4(2):e4598. doi: 10.1371/journal.pone.0004598 (PMC2643479; doi:10.1371/journal.pone.0004598)
Supplement: Table S3 — Sequence variation and species identification ability of two plastid regions in Hordeum (all 32 species). No length variation is observed among the sequences. GenBank acc. nos. EU118371-EU118422, EU118427-EU118478. (0.04 MB DOC) [file pone.0004598.s004.doc]

| **Region(s)** | **Sequence length** | **Variable sites** | **Unique species**  (%) |
| --- | --- | --- | --- |
| *matK* | 797 | 29 | 13 (41%) |
| *rpoC1* | 575 | 9 | 5 (16%) |
| *matK*+*rpoC1* |  |  | 15 (47%) |
