# Supplementary material for: How Many Loci Does it Take to DNA Barcode a Crocus?
Source: PLoS One. 2009 Feb 25;4(2):e4598. doi: 10.1371/journal.pone.0004598 (PMC2643479; doi:10.1371/journal.pone.0004598)
Supplement: Table S4 — Sequence variation and species identification ability of six plastid regions in Scalesia (5 of 15 species). No length variation is observed among the sequences. GenBank acc. nos. EU118423-EU118426, EU118479-EU118483, EU118494-EU118498, EU118509-EU118513, EU118524-EU118527, EU118536-EU118539. (0.04 MB DOC) [file pone.0004598.s005.doc]

| **Region** | **Sequence length** | **Variable sites** | **Unique species**  (%) |
| --- | --- | --- | --- |
| *matK* | 820 | 0 | 0 |
| *rpoC1* | 530 | 0 | 0 |
| *ndhJ* | 374 | 0 | 0 |
| *rpoB* | 499 | 0 | 0 |
| *ycf5* | 383 | 0 | 0 |
| *accD* | 367 | 0 | 0 |
